# Opposing Patterns of Spatial Synchrony in Lyme Disease Incidence

**DOI:** 10.1007/s10393-024-01677-8

**Published:** 2024-05-04

**Authors:** Asad E. Ali, Allison M. Gardner, Herman H. Shugart, Jonathan A. Walter

**Affiliations:** 1 Department of Environmental Sciences, University of Virginia, 291 McCormick Road, Charlottesville, VA 22903 USA; 2 Alabama College of Osteopathic Medicine, 445 Health Sciences Boulevard, Dothan, AL 36303 USA; 3 School of Biology and Ecology, University of Maine, 5722 Deering Hall, Orono, ME 04469 USA; 4 Center for Watershed Sciences, University of California, 1 Shields Ave, Davis, CA 95616 USA

**Keywords:** *Borrelia burgdorferi*, Synchrony, Weather, Land use, Human health

## Abstract

Incidence of Lyme disease, a tick-borne illness prevalent in the US, is increasing in endemic regions and regions with no previous history of the disease, significantly impacting public health. We examined space–time patterns of Lyme disease incidence and the influence of ecological and social factors on spatial synchrony, i.e., correlated incidence fluctuations across US counties. Specifically, we addressed these questions: Does Lyme disease incidence exhibit spatial synchrony? If so, what geographic patterns does Lyme disease synchrony exhibit? Are geographic patterns of disease synchrony related to weather, land cover, access to health care, or tick-borne disease awareness? How do effects of these variables on Lyme disease synchrony differ geographically? We used network analysis and matrix regression to examine geographical patterns of Lyme disease synchrony and their potential mechanisms in 399 counties in the eastern and Midwestern US. We found two distinct regions of synchrony in Northeast and upper Midwest regions exhibiting opposing temporal fluctuations in incidence. Spatial patterns of Lyme disease synchrony were partly explained by land cover, weather, poverty, and awareness of tick-borne illness, with significant predictive variables changing regionally. However, the two regions may have become more synchronous over time, potentially leading to higher-amplitude nation-wide fluctuations in disease incidence.

## Introduction

In biological systems such as the dynamics of animal populations or disease incidence, temporal fluctuations are commonly correlated across many locations, a phenomenon known as spatial synchrony (Liebhold et al. 2004; Walter et al. 2017). Synchrony can be caused by a variety of different mechanisms, including the “Moran effect,” in which spatially correlated environmental effects, such as climate and weather variation, impart synchrony through their influence on biological processes, dispersal, or interactions with synchronized species (Liebhold et al. 2004). Synchrony plays a key role in large-scale ecological and epidemiological dynamics as fluctuations that are shared across locations are amplified in the aggregate, and studies of synchrony have provided new insights into how environmental drivers shape biological dynamics (Koenig & Liebhold 2016; Walter et al. 2017). Recent studies have emphasized that synchrony often exhibits complex geographic patterns that reflect spatiotemporal patterns of underlying drivers of synchrony or of mechanisms that modify the effects of underlying drivers on local populations (Walter et al. 2017). An emerging approach to studying geographies of synchrony envisions a network of locations linked by synchronous dynamics and investigates the structure of these networks to understand the nature and causes of synchrony (Walter et al. 2017; Anderson et al. 2018; Moustakas et al. 2018).

Synchrony is a prominent feature of disease outbreaks such as whooping cough, measles (Rohani 1999), and influenza. Viboud et al. (2006) found high levels of synchrony in flu outbreaks in urbanized, densely populated areas in the US, such as cities including New York and Los Angeles. In a plant disease system, Penczykowski et al. (2015) found increasing synchrony in the fungal pathogen *Podosphaera plantaginis* as plant exposure to freezing decreased during the period of 2001–2013. Thus, spatial synchrony can be observed not only in animal and plant populations, but also disease outbreaks which affect plant, animal, and human populations. However, spatial synchrony in diseases of humans has mainly been studied in highly contagious viruses and bacteria, rather than pathogens arising from animal-vectored pathogens, and so investigations have naturally focused on transmission networks, as opposed to ecological effects of weather and habitat conditions.

Lyme disease is a tick-borne illness caused by infection of the *Borrelia burgdorferi* bacterium, incidence of which is increasing through time and expanding into new areas (Schwartz et al. 2017; Bisanzio et al. 2020; Gardner et al. 2020). According to the US Centers for Disease Control and Prevention (CDC), an estimated 476,000 people are treated for Lyme disease each year (2021). The geographic range where Lyme disease cases are likely locally acquired has expanded over time from its initial core in the northeastern US (Bisanzio et al. 2020; Eisen et al. 2016; Gardner et al. 2020; Kugeler & Eisen 2020). Today, areas of high incidence are concentrated in the Northeast and upper Midwest (Schwartz et al. 2017); in both regions, the number of counties with high incidence of Lyme disease expanded dramatically (> 320% and ≈250%, respectively; Kugeler et al. 2015).

However, much remains unknown about large-scale spatiotemporal patterns and drivers of Lyme disease incidence. Lyme disease transmission is affected by a wide variety of factors, including the pathogen life cycle, pathogen–vector and vector–host ecology (Levi et al. 2012, 2015; Halsey et al. 2018; Ostfeld et al. 2018), habitat and land cover change (Wood & Lafferty 2013; Kilpatrick et al. 2017; Conte et al. 2021; Diuk-Wasser et al. 2021; López-Pérez et al. 2021; VanAcker et al. 2019), climate and climate change (Burtis et al. 2016; Ostfeld & Brunner 2015; Brownstein et al. 2005; Kotchi et al. 2021), and social factors that may predispose certain populations for a higher risk of contracting Lyme disease (Couper et al. 2021; Scott & Scott 2018; Springer and Johnson 2018). Despite considerable effort into resolving the ecological and epidemiological factors shaping Lyme disease dynamics, whether Lyme disease incidence exhibits spatial synchrony, and if so what the patterns and drivers are, is to our knowledge unknown. Resolving these questions is consequential given the importance of synchrony for determining regional-scale disease dynamics.

The purpose of this study is to determine if spatiotemporal patterns in Lyme disease occurrence exhibit spatial synchrony, and the patterns and drivers of spatial synchrony in Lyme disease in the US. We focus on the following research questions: (1) Does Lyme disease incidence exhibit spatial synchrony, and if so, what geographic patterns does Lyme disease synchrony exhibit? (2) Are geographic patterns of Lyme disease synchrony related to weather, land cover, access to health care, or awareness of tick-borne disease? (3) How do the effects of these variables on Lyme disease synchrony differ geographically? By answering these questions and establishing links between the spread of Lyme disease with land cover, climate, and socioeconomic phenomena, we can better contextualize the complex spatiotemporal patterns of a disease which, along with affecting hundreds of thousands of Americans each year, exhibits ecologically and epidemiologically distinct trends across the range of endemic regions in the US.

## Methods

We used multiple analyses to characterize the spatial synchrony of Lyme disease incidence and infer potential mechanisms. We first used network analysis to examine geographies of Lyme disease synchrony in our study region (Walter et al. 2017), focusing on identifying groups of U.S. counties having synchronous temporal patterns of Lyme disease incidence. We then tested whether land cover characteristics, weather, health care access, and awareness of tick-borne illness explain geographic patterns of spatial synchrony in Lyme disease incidence.

### Data

We determined county-level Lyme disease incidence (reported cases per 1000 people) across US counties by obtaining annual 2000–2018 Lyme disease case data from the US Centers for Disease Control and Prevention (CDC 2021) and annual aggregated population time series from the US Census Bureau (USCB 2021). Annual case counts were normalized by county population in that year. To satisfy requirements of our statistical tests, we removed all counties which had three or more years of zero reported cases, and because we were interested in places where Lyme disease is not rare, we omitted counties with time-averaged incidence rates below 1 in 100,000 people. Counties meeting this criterion were overwhelmingly located in the Great Lakes region and in the eastern USA from North Carolina northward, so we also removed from our dataset the small number of geographically isolated counties, such as in California, outside these regions. Additionally, in the western US, Lyme disease is transmitted by a different vector, *Ixodes pacificus* (MacDonald et al. 2017).

We obtained land cover data (2011 conditions; 30-m spatial resolution) from the National Land Cover Database (NLCD 2021) and used it to compute the percent forest, percent developed, and density of forest edges (length of forest edge per county area; m/ha) for each county. Percent forest is the total percent (by area) of each county comprised of the Deciduous Forest, Evergreen Forest, and Mixed Forest NLCD classes. Percent developed is the total percent (by area) of each county comprised of the Developed–Open Space, Developed–Low Intensity, Developed–Medium Intensity, and Developed–High Intensity NLCD classes. Although *I. scapularis* is more strongly associated with deciduous broadleaf than evergreen needleleaf forests (Ginsberg et al. 2004), deciduous and mixed forest made up 85% of forest in our study area, so for simplicity we considered all forest types together. We used data from PRISM (NACSE 2020) to quantify weather variables thought to influence the population dynamics of Lyme disease vectors or hosts (Burtis et al. 2016; Ostfeld & Brunner 2015; Brownstein et al. 2005; Kotchi et al. 2021). PRISM data are available at 4 km spatial resolution, and temperature time series were obtained from the spatial centroid of each county. We computed the number of hot, dry days (T > 25 °C, precipitation = 0) per year, taken over the full year and in shorter phenological windows based on the life cycle of blacklegged ticks. To correspond generally with the nymphal life stage, we summed the number of hot, dry days from May through July, while to correspond generally with the larval life stage we summed the number of hot, dry days from August through September (Burtis et al. 2016). Additionally, we computed the annual time series of average winter (January through March) minimum temperature. Following findings by Couper et al. (2021), we also considered two human population variables: the percentage of residents living in poverty (averaged over 2000–2018; USCB 2021), and temporal patterns in the frequency Google searches for “tick” from 2004 (the first year data were available) to 2017 reported by Google Trends. The latter were available at the level of Designated Metropolitan Areas (DMAs), and for our analyses, the time series for a DMA was considered to apply to all counties comprising it. Like Couper et al. (2021), we considered poverty, the percentage of county population living under the poverty line, an index of access to healthcare and Google searches an index of public awareness of tick-borne illness.

Prior to analysis, all-time series were linearly detrended, transformed to approximate normality using a Box-Cox procedure, and scaled to have unit variance.

### Analyses

We first used a network modularity analysis to identify groups of counties having relatively high within-group spatial synchrony but relatively weak between-group synchrony of spatially synchronous counties (Newman 2006). We constructed a weighted synchrony network by quantifying spatial synchrony in Lyme disease incidence among all pairs of counties using Pearson correlation. Pearson correlation is a standard measure of synchrony (Liebhold et al. 2004; Walter et al. 2017) ranging − 1 to 1; positive values correspond to synchrony. We then used an elaboration of the algorithm of Newman (2006) to networks with positive and negative weights (Walter et al. 2021) to detect network modules. This algorithm has a built-in stopping criterion that acts as a test of whether subdivisions of the dataset have statistical support.

We next used matrix regression on similarity matrices (MRM; Lichstein 2007; Haynes et al. 2013) to assess evidence for potential weather, land cover, and human population mechanisms of geographic variation in spatial synchrony. MRM tests the hypothesis that the structure of a response matrix is more similar than expected by chance to that of one or more predictor matrices, while controlling for effects of other predictor matrices. Thus, MRM examines evidence for likely drivers of synchrony and for mechanisms that do not cause synchrony per se but instead modify its strength based on statistical similarity of geographic pattern. As above, spatial synchrony in Lyme disease incidence was represented as the matrix of all pairwise Pearson correlation coefficients among county-level Lyme disease incidence time series. Predictors considered included: spatial synchrony in hot, dry days (all year, nymphal period, larval period); spatial synchrony in winter average minimum temperature; spatial synchrony in Google searches for “tick”; similarity in percent forest land cover; similarity in percent developed land cover; similarity in forest edge density; similarity in percent of residents in poverty; and spatial proximity. In all cases, synchrony was measured using pairwise Pearson correlation. Similarities were transformations of pairwise Euclidean distances using the equation similarity = 1 − (*d*_*ij*_/max(*d*_*ij*_)), where *d* is the Euclidean distance in whatever metric between counties *i* and *j*. MRM can equivalently be applied to distance matrices, or a mix of distance and similarity matrices, but since synchrony is a kind of similarity, we expressed all other variables as similarities to aid interpretation of regression coefficients.

We used MRM to examine the relationships between Lyme disease synchrony and these predictors, considering first all studied counties together, and subsequently each of the modules identified in our first analysis, separately. One reason that spatial synchrony, generally, might show distinct geographic patterns is that different drivers tend to predominate in different places (Walter et al. 2017). Since some predictors were collinear and had similar interpretations, we tested six candidate models and selected the one having the highest *R*^2^. Each model contained spatial proximity, one (out of three) hot, dry days synchrony variable, winter minimum temperature synchrony, similarity in percent forest or percent developed, similarity in forest edge density, similarity in poverty percentage, and synchrony in web searches for “tick.” Statistical significance of model terms is evaluated using a permutation-based pseudo-*t* test and a permutation-based *F*-test provides a test that the model produces better predictions than randomness (Legendre et al. 1994). Statistical significance was assessed at the α = 0.05 level.

Analyses were conducted in R version 4.0.3 using the ‘wsyn’ (Reuman et al. 2021) and ‘ecodist’ (Goslee & Urban 2007) packages.

## Results

**Fig. 1:**
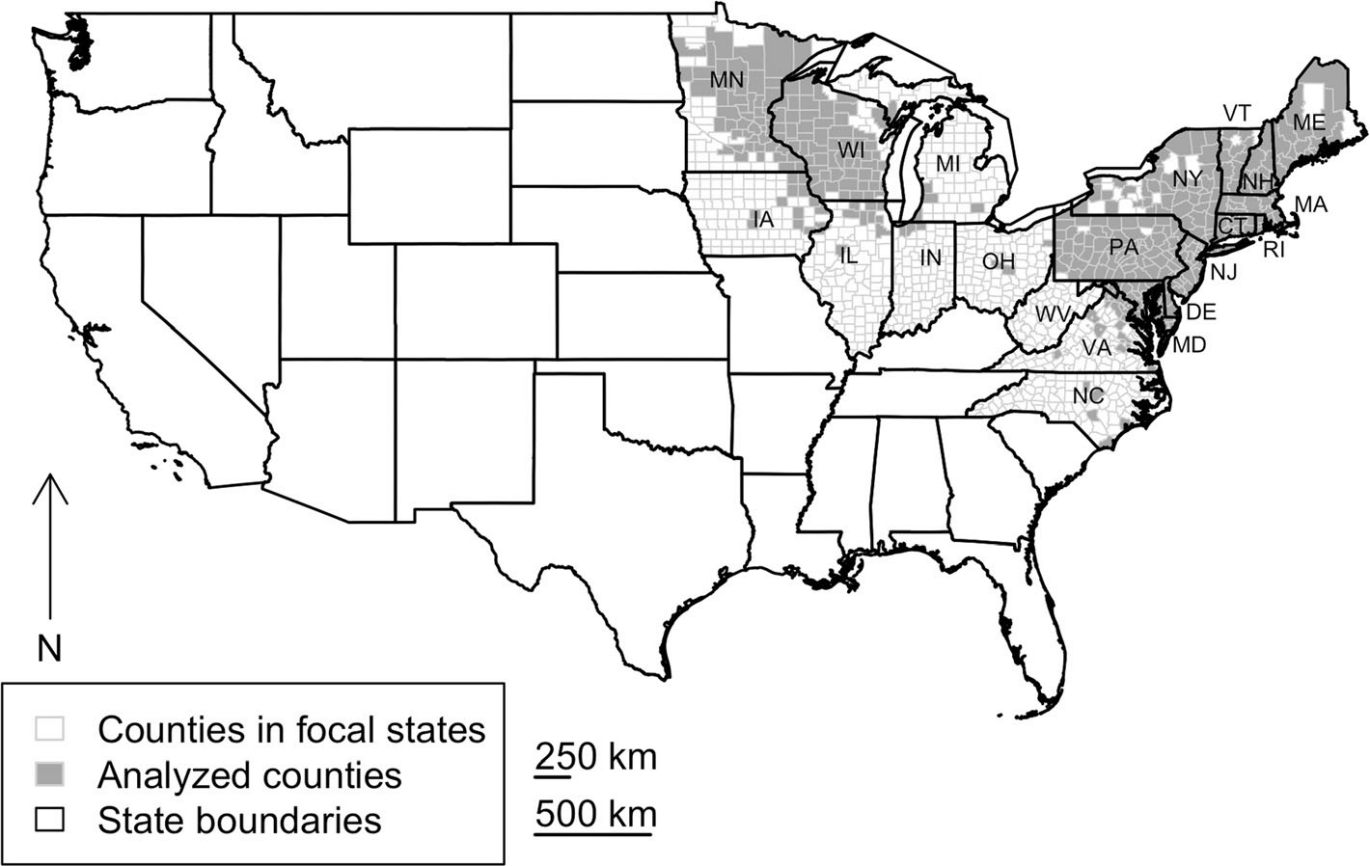
Map of study area in conterminous USA. Code to state abbreviations: CT, Connecticut; DE, Delaware; IA, Iowa, IL, Illinois, IN, Indiana, OH, Ohio, PA, Pennsylvania, ME, Maine, MD, Maryland, MA, Massachusetts, MI, Michigan, MN, Minnesota, NH, New Hampshire, NJ, New Jersey, NY, New York, NC, North Carolina, RI, Rhode Island, VA, Virginia, WV, West Virginia, WI, Wisconsin.

Our analysis focused on 416 counties meeting our inclusion criteria, located in the states of Connecticut, Delaware, Iowa, Illinois, Indiana, Ohio, Pennsylvania, Maine, Maryland, Massachusetts, Michigan, Minnesota, New Hampshire, New York, North Carolina, Rhode Island, Virginia, West Virginia, and Wisconsin (Fig. 1). From 2000 to 2018, county-level mean annual Lyme disease incidence ranged from 0.01 to 6.4 cases per 1000 people. Interannual variability (standard deviation) in incidence ranged from 0.007 to 3.9 cases per 1000 people.

**Fig. 2:**
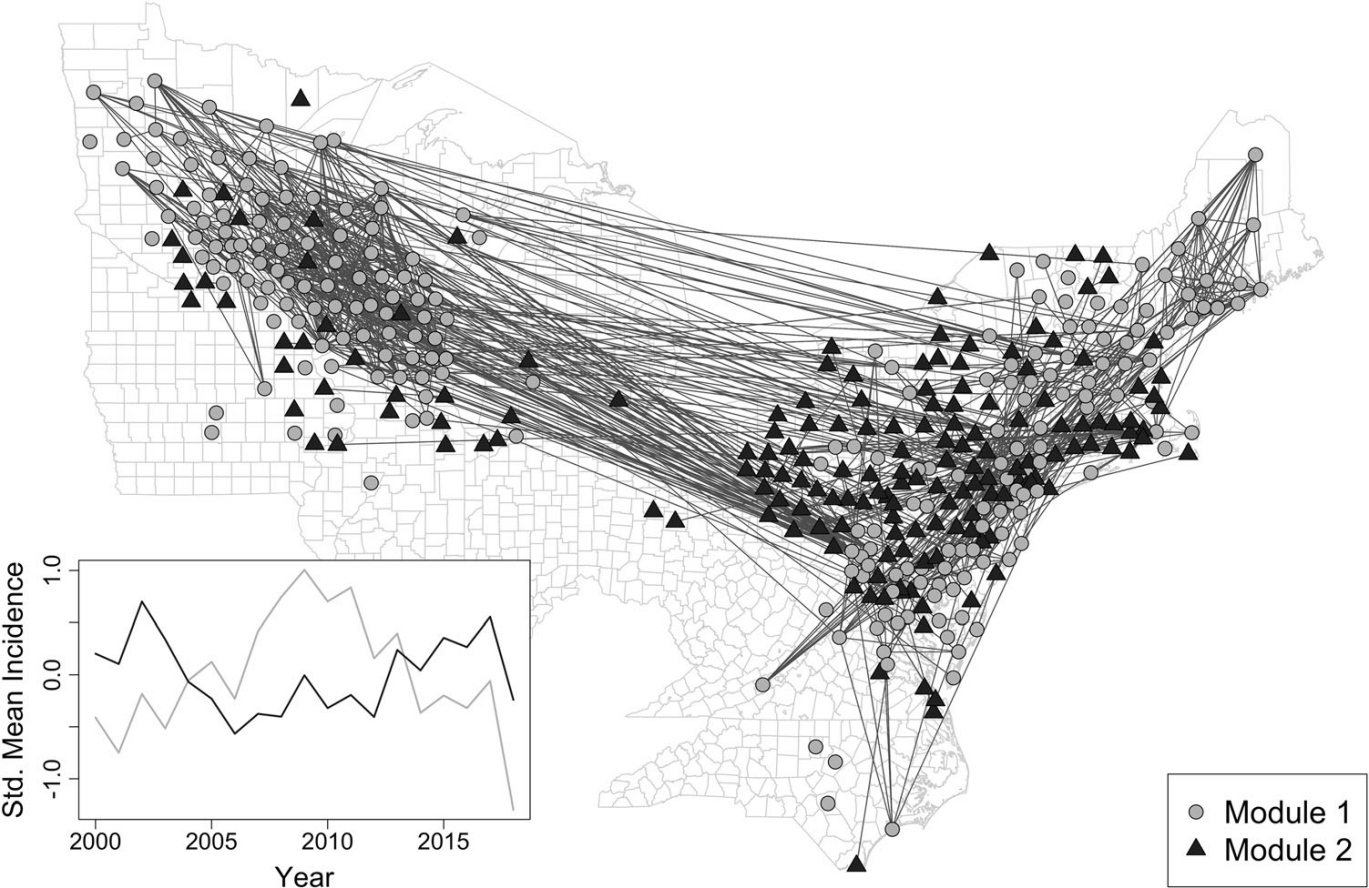
The spatial synchrony of Lyme disease. Lyme disease exhibits two clusters having distinct dynamics; the edges and nodes depicted represent the strongest 1% correlations among the counties. In the inset, Lyme disease incidence time series were standardized to have mean = 0 and unit variance and averaged within module.

Modularity analysis detected two distinct modules in Lyme disease spatial synchrony corresponding to groups of counties with relatively high within-group and low between-group synchrony (Fig. 1). Module 1 (*n* = 235 counties) was concentrated primarily in the upper Midwestern region of the US, while Module 2 (*n* = 181 counties) was concentrated primarily in the northeastern region; both of these modules displayed strong intra-module synchrony (visually apparent in the relatively high number of links connecting gray nodes to gray nodes and black nodes to black nodes) but low inter-module synchrony (visually apparent in the lack of links connecting gray nodes and black nodes). Corroborating that intra-module synchrony was greater than inter-module synchrony, the median spatial synchrony (Pearson correlation) between counties in the same module was 0.26 (inter-quartile range = 0.08–0.46) and the median spatial synchrony (Pearson correlation) between counties in different modules was − 0.06 (inter-quartile range = − 0.25 to 0.13). We also found that Modules 1 and 2 exhibited opposite patterns in mean incidence over time, demonstrating quasi-cyclical patterns which reached peaks and troughs at opposite points in time until ≈2014 (Fig. 1; Pearson correlation = − 0.43) (Fig. 2).

In our MRM analysis of all studied counties, the regression model most predictive of synchrony in Lyme disease (by *R*^2^) represented summer temperatures as hot, dry days during the larval period and land cover composition as percent forest. Spatial synchrony in Lyme disease was positively related to spatial synchrony in awareness of tick-borne disease (Google searches for “ticks”) and similarity in percent forest (Table 1); other effects were not statistically significant at the α = 0.05 level. The model *R*^2^ was 0.008, but low *R*^2^ values for MRM models are common (Walter et al. 2017; Anderson et al. 2018) and a highly significant test for lack of fit (*p* < 0.001) allows us to reject the null hypothesis that the model is not predictive of geographical patterns of spatial synchrony.

**Table 1:** MRM Analysis, Spatial Synchrony in All Counties.

Variable	Coefficient	*p* value
Spatial proximity	0.187	0.061
Larval hot, dry days synchrony	− 0.043	0.119
Winter avg. min. temperature synchrony	− 0.022	0.809
Edge density similarity	− 0.046	0.089
Percent forest similarity	0.044	*0.010*
Poverty rate similarity	− 0.004	0.909
Google tick search synchrony	0.094	*0.005*

*R*^2^ = 0.008, model *p* value < 0.001.

Variables with *p*-value < 0.05 are italicized.

In our MRM model most predictive of Lyme disease synchrony by *R*^2^ for Module 1, which was concentrated in the upper Midwest states, summer temperatures were represented as hot, dry days of the nymphal phase, while the composition of land cover was represented as percent developed. Spatial synchrony of Lyme disease was positively associated with spatial synchrony in poverty, Google searches, and nymphal hot, dry days, while other effects were not found to be statistically significant (Table 2). The model *R*^2^ was 0.047, but the significant test for lack of fit (*p* < 0.001) for this model also allows us to reject the null hypothesis that the model is not predictive of geographical patterns of spatial synchrony.

**Table 2:** MRM Analysis, Spatial Synchrony in Module 1 Counties.

Variable	Coefficient	*p* value
Spatial proximity	− 0.017	0.807
Nymphal hot, dry days synchrony	0.088	*0.015*
Winter avg. min. temperature synchrony	0.210	0.119
Edge density similarity	0.010	0.748
Percent developed similarity	− 0.039	0.285
Poverty rate similarity	0.149	*0.006*
Google tick search synchrony	0.107	*0.006*

*R*^2^ = 0.047, model *p* value < 0.001.

Variables with *p*-value < 0.05 are italicized.

Lastly, our best model for Module 2 counties had an *R*^2^ of 0.023 and represented summer temperatures as hot, dry days of the larval period and land cover composition as percent developed (Table 3). In this model, synchrony in geographical proximity was significantly positively associated with Lyme disease synchrony, while synchrony in winter average minimum temperature was negatively associated with synchrony in Lyme disease. Again, given that the model *p* < 0.001, we rejected the null hypothesis that the model is not predictive of geographical patterns of spatial synchrony.

**Table 3:** MRM Analysis, Spatial Synchrony in Module 2 Counties.

Variable	Coefficient	*p* value
Spatial proximity	0.305	*0.025*
Larval hot, dry days synchrony	0.064	0.087
Winter avg. min. temperature synchrony	− 0.210	*0.022*
Edge density similarity	0.038	0.275
Percent developed similarity	0.036	0.284
Poverty rate similarity	0.029	0.505
Google tick search synchrony	− 0.002	0.972

*R*^2^ = 0.023, model *p*-value < 0.001.

Variables with *p*-value < 0.05 are italicized.

## Discussion

We found that the geography of spatial synchrony of Lyme disease from 2000 to 2018 could be characterized by two distinct regions, the Northeast and upper Midwest, that exhibited opposing temporal fluctuations in Lyme disease incidence (Q1); though the northernmost portion of the Northeast in Maine exhibited trends in incidence that correlated more strongly with the upper Midwest (Q1). Prior studies have found that Lyme disease expanded into Maine later than the rest of the Northeast (Elias et al. 2021); this may be a potential mechanism of the similar trends seen in this region and the overall upper Midwest. We found that the spatial structure of Lyme disease synchrony was related to land cover, winter and summer temperatures, access to health care, and awareness of tick-borne illness (Q2); however, the influence of these variables differed among regions (Q3). For example, in the upper Midwest, (Module 1) increase in Lyme disease incidence was positively associated with nymphal-stage hot and dry days, increased poverty, and Google searches for “ticks.” In the Northeast (Module 2), however, Lyme disease incidence was positively associated with geographical proximity and negatively correlated with winter average minimum temperatures. These results shed light on the broader spatiotemporal patterns of Lyme disease in the US; moreover, they suggest that the most important drivers of Lyme disease incidence differ regionally.

Our findings regarding the geography of Lyme disease spatial synchrony in the US aid in contextualizing prior studies indicating the concentration of Lyme disease cases in the Northeastern and upper Midwestern regions (Schwartz et al. 2017) by emphasizing the differences in incidence trends in these regions. Specifically, our study sheds light on the opposing patterns of Lyme disease incidence and spatial synchrony in the Northeast and upper Midwest regions of the US. There was an apparent cyclical pattern in disease incidence in both modules that had Lyme disease incidence in the two regions fluctuating in opposition through the first ¾ of the study period. Intriguingly, however, since ≈2014 the mean time series for each module appear to be more synchronized. The geographic range where Lyme disease cases are likely locally acquired has expanded over time from its initial core in the northeastern US (Bisanzio et al. 2020; Eisen et al. 2016; Gardner et al. 2020; Kugeler & Eisen 2020). Following invasion of a new area, there may be a lag time before the newly established population becomes synchronized with longer-established populations, with rates of synchronization depending on strength of endogenous population regulation (Bjørnstad et al. 2008). If indeed formerly anti-synchronous regions of relatively high Lyme disease prevalence have become synchronized, this would have major implications for the year-to-year variance in total Lyme disease cases in the USA, as anti-synchronous fluctuations tend to cancel in the sum, while synchronous fluctuations reinforce each other.

Two classical mechanisms of spatial synchrony are synchronous weather variation and dispersal, which commonly manifest in part as declines in synchrony with increasing distance (Liebhold et al. 2004). Consistent with this expectation, we tended to find that nearby counties had more synchronous Lyme disease incidence. We also found that disease synchrony was positively correlated with synchrony in nymphal-stage hot, dry days in the upper Midwest, and in the Northeast a positive association between Lyme disease synchrony and synchrony in larval-stage hot, dry days approached statistical significance (Tables 2, 3). Thus, synchrony in summer weather partly explains synchrony in Lyme disease incidence. In the Northeast, we also found a negative association between Lyme disease synchrony and synchrony in winter minimum temperatures. Although some degree of decoupling between winter weather and Lyme disease dynamics could be expected given the ability of *I. scapularis* to survive cold ambient temperatures due to insulation in microhabitats, such as snowpacks (Linske et al. 2019), the reason for a negative association is unclear and could possibly reflect type-1 error or correlation with some third, unmeasured variable having a mechanistic effect on Lyme disease dynamics. Aspects of winter weather unaddressed by this study such as temperature variation and precipitation may also play a role in Lyme disease dynamics (Subak 2003).

Prior studies have highlighted the significance of forest land cover and human development on the transmission of Lyme disease (Kilpatrick et al. 2017, Wood & Lafferty 2013, Conte et al. 2021, Diuk-Wasser et al*.* 2020). For example, that Lyme disease incidence may be positively associated with land-use change and forest fragmentation (Allan et al. 2003; Brownstein et al. 2005; Kilpatrick et al. 2017). While we did not find forest edge density (a metric of forest fragmentation) to be predictive of Lyme disease synchrony, in our analysis across all counties, we found that Lyme disease was more synchronous between counties with a similar percentage of forest land cover (Table 1). In our module-specific (regional) analyses, similarity in percentages of developed land was a better predictor of disease synchrony than similarity in percent forest cover, although these were not statistically significant predictors.

In our analysis of socioeconomic variables, we found that Google searches for “ticks” were significant determinants of Lyme disease synchrony in our analysis of all counties and the upper Midwest (Module 1). This finding is supported by a prior study by Couper et al. (2021), who found that Lyme disease incidence was positively associated with Google searches, used as a proxy for disease awareness among the public. Unlike other variables we considered, awareness of tick-borne disease is not an ecological mechanism of synchrony or its geography; rather, encounters with ticks likely drive variation in web searches. Still, we included this variable in our analyses to help constrain variation possibly arising if awareness influences rates of diagnosis. In contrast to results reported by Couper et al. (2021), who noted no significant effect of poverty on disease incidence, we found that poverty was positively associated with disease synchrony in the upper Midwest (Module 1). Given the sociological complexities of poverty, a wide variety of factors such as lower insurance coverage, the decreased affordability of healthcare, and subsequent underdiagnoses could be potential mechanisms of the effect of poverty on disease synchrony. Relatedly, Springer and Johnson (2018) found disease incidence to be associated with higher-income areas as well as areas with a higher percentage of vacant housing units. Taken together, our results underscore the importance of considering both ecological and socioeconomic factors in understanding the dynamics of human diseases with animal reservoirs and vectors.

An aspect of Lyme disease ecology that we did not address in this study was the effects of white-tailed deer and white-footed mouse populations on disease transmission. Addressing this variable in our analysis would have been difficult due to the scarcity of high-resolution, county-level data on the populations of these hosts. High mouse population density has been strongly correlated with Lyme disease risk (LoGiudice et al. 2003); additionally, the 4-year population cycle of white-footed mice has been found to have a strong impact on tick-borne pathogen transmission (Wang et al. 2009). Furthermore, white-tailed deer populations have functioned as hosts to amplify *I. scapularis* reproduction, increasing the risk of *B. burgdorferi* transmission (Wood & Lafferty 2013). Although to our knowledge data on matching spatiotemporal scales do not exist to use in our analyses, there is evidence that white-footed mouse population cycles are spatially synchronous (Haynes et al. 2009), possibly contributing to spatial synchrony in Lyme disease by transmitting synchrony through host–pathogen interactions.

Our study assumed that the Lyme disease data were compiled in a relatively uniform, consistent manner with minimal discrepancies from county to county. This may not be the case in reality. While the CDC continues to organize disease data annually, the sociopolitical determinants of Lyme disease and public health reporting may have caused noise and bias in our data. Lyme disease prevention and reporting have been strongly associated with awareness of the disease in the northeastern and upper Midwestern regions, where the disease is endemic in the US (Herrington et al. 1997); it may be possible, therefore, that underdiagnoses of the disease in previously non-endemic regions may have skewed our data on the geography of Lyme disease synchrony and incidence, especially in regard to the lower number of cases in the mid-Atlantic region. Furthermore, healthcare providers’ assumptions of patients being infected with Lyme disease without taking a test for the disease may also cause more cases to go unreported. Additionally, it is possible that noise in our data due to reporting inconsistencies contributed to the lower *R*^2^ values across our models.

Spatial synchrony is well-known to manifest in disease outbreaks, but prior work has focused mainly on viral and bacterial infections that are directly transmitted between infected hosts, such as influenza and whooping cough (Rouhani et al*.* 1999, Viboud et al. 2006, Moustakas et al. 2018), and hence, these studies have focused primarily on movements of infected hosts as mechanisms of synchrony. In our vector-borne study system, we found evidence that weather may synchronize Lyme disease dynamics across regional scales, and also that spatial habitat conditions may modify synchrony and contribute to its spatial structure. This is consistent with the importance of vectors and alternate hosts to the dynamics of vector-borne diseases. Similar classes of mechanisms likely shape synchronous dynamics of other vector-borne diseases. While to our knowledge this seems little studied, the incidence of vector-borne diseases may still fluctuate over orders of magnitude, and understanding the large-scale drivers of such fluctuations can improve preparedness of public health systems and potentially suggest interventions to prevent or mitigate large-scale outbreaks.

Given these patterns of weather potentially shaping Lyme disease synchrony across the Northeast and upper Midwest, our study may help in shedding light in how broader climatic patterns such as climate change may result in a more synchronized rise in incidence across these geographic regions, further building on prior studies such as those conducted by Burtis et al. (2016), Ostfeld & Brunner (2015), Brownstein et al. (2005), and Kotchi et al. 2021.
